# Case of Severe Neuralgic Affection, Cured by Carbonate of Iron

**Published:** 1826-12

**Authors:** 


### NEURALGIA.

### *Case of severe Neuralgic Affection, cured by Carbonate of Iron.*

THE following is a copy of a letter from Dr. JAQUES, of Harrowgate, to Mr. HUTCHINSON, of Southwell.

I have been favoured with your letter, and shall be happy to give you every information in my power respecting Mr. C. M.'s case.

I was consulted by him on the 30th of July in the present year. I found him confined to his bed by a violent pain in his head. Upon questioning him very minutely about the nature of his complaint, and the symptoms attending it, he gave me the following account, as near as I can recollect:—The disease he was then suffering from he had laboured under for more than seven years. It generally commenced its attack by a violent pain in the left temple, which remained frequently for twenty-four hours, and then passed over the head into the right temple: the pain often remained three days and nights, with very great violence,—sometimes only a few hours. It left him suddenly, and he was then quite well. He was of a very spare habit of body, and of great delicacy of constitution. The medical gentleman he consulted at Belfast bled, blistered, cupped, and leeched him, and kept him on very low diet; from which treatment he derived no benefit. He went to Dublin about seven years since, and consulted Dr. PERCIVAL, under whose care he remained some time: he at last candidly told him he could not prescribe any thing likely to relieve him, and with equal candour announced to his patient the probability of a fatal issue. He took the advice of several other medical gentlemen: various medicines were tried,—mercury, camphor, opium, internally and externally. Arsenic was the only medicine that ever had any effect, and it certainly did arrest it for a very short time. One gentleman had prescribed iron, but in very small doses, which gave no relief. He then went to Edinburgh, where he consulted Dr. THOMPSON, who was of opinion that tape-worm was the cause, and gave him large doses of turpentine; from which not deriving the smallest benefit, he desired him to come to this place. He made a trial of the water for some time, but obtained no relief; and he consequently consulted me on the 30th of July.

I immediately, having your excellent plan in view, ordered him twenty grains of the Subcarbonate of Iron three times a-day, and gradually increased the dose. On the 20th of August he took fifty grains three times a-day, in which he persevered so long as he remained here: at the same time, he drank five half-pints of the chalybeate water every day.

Mr. C. M. never had a return of the complaint after the first five days. When he left us, he had been quite well more than a month, and before this period he never enjoyed an intermission of more than a very few days. His brother, who remained here, had a letter from him, saying that, in returning home, he had been up two nights travelling, but had suffered nothing from his complaint. His strength was also much improved, and he was able to go through more fatigue than he was capable of before.

Two days ago I received a letter from my patient, from which the following is an extract:—

"In compliance with your desire, so kindly expressed, that I should write to acquaint you with the state of my health, I have the great pleasure of saying that, since I had the benefit of your aid at Harrowgate, I have (thank God!) enjoyed an almost entire freedom from pain; and have progressively increased in weight and strength since I had the pleasure of seeing you. My brother, on his return, informed me I should discontinue taking the iron: this I think had a very good effect; and since that time I have not tasted medicine of any sort. My friends here (medical included) consider my recovery almost miraculous."

I suppose you never saw a stronger instance of the value of your discovery. The length of time, exceeding seven years,—the violence of the attacks,—the variety and inutility of the medicines administered, and also the great number of eminent men he had in vain consulted,—all conspire to confirm, on the most solid basis of experience, the very great powers of the remedy which you have so laudably pointed out.

